# Obesity and colorectal cancer: molecular features of adipose tissue

**DOI:** 10.1186/s12967-016-0772-5

**Published:** 2016-01-22

**Authors:** Javier Martinez-Useros, Jesus Garcia-Foncillas

**Affiliations:** Translational Oncology Division, Oncohealth Institute, FIIS-Fundacion Jimenez Diaz, Av. Reyes Catolicos 2, 28040 Madrid, Spain

**Keywords:** Colorectal cancer, Obesity, Overweight, BMI, Adipose tissue, Cytokines, Inflammation, 4-HNE, MDA, MPE

## Abstract

The huge part of population in developed countries is overweight or obese. Obesity is often determined by body mass index (BMI) but new accurate methods and ratios have recently appeared to measure body fat or fat located in the intestines. Early diagnosis of obesity is crucial since it is considered an increasing colorectal cancer risk factor. On the one hand, colorectal cancer has been strongly associated with lifestyle factors. A diet rich in red and processed meats may increase colorectal cancer risk; however, high-fiber diets (grains, cereals and fruits) have been associated with a decreased risk of colorectal cancer. Other life-style factors associated with obesity that also increase colorectal cancer risk are physical inactivity, smoking and high alcohol intake. Cutting-edge studies reported that high-risk transformation ability of adipose tissue is due to production of different pro-inflammatory cytokines like IL-8, IL-6 or IL-2 and other enzymes like lactate dehydrogenase (LDH) and tumour necrosis factor alpha (TNFα). Furthermore, oxidative stress produces fatty-acid peroxidation whose metabolites possess very high toxicities and mutagenic properties. 4-hydroxy-2-nonenal (4-HNE) is an active compounds that upregulates prostaglandin E2 which is directly associated with high proliferative colorectal cancer. Moreover, 4-HNE deregulates cell proliferation, cell survival, differentiation, autophagy, senescence, apoptosis and necrosis via mitogen-activated protein kinase (MAPK), phosphoinositide 3-kinase (PIK3CA)—AKT and protein kinase C pathways. Other product of lipid peroxidation is malondialdehyde (MDA) being able to regulate insulin through WNT-pathway as well as having demonstrated its mutagenic capability. Accumulation of point mutation enables genomic evolution of colorectal cancer described in the model of Fearon and Vogelstein. In this review, we will summarize different determination methods and techniques to assess a truthfully diagnosis and we will explain some of the capabilities that performs adipocytes as the largest endocrine organ.

## Background

In western countries a huge part of population is overweight or obese, for example in the US more than 30 % of the population belong to this group [1]. In the UK obesity is considered the second cancer risk-factor after smoking [2]. Obesity refers a disorder that involves excessive accumulation of body fat and thereof, raising the risk of metabolic syndrome. Obesity is one of the risk factors such as gender, race, dietary habits or smoking history that has been linked to the most relevant cancers like breast [3], gynaecological [4], liver [5] and colorectal cancer (CRC) [6]. Colorectal cancer is one of the most common gastrointestinal malignant tumours in the world and presents one of the highest rates of morbidity and mortality worldwide [7]. In addition, colorectal cancer-obesity relationship is reinforced by the importance of nutrition in this cancer. Thus, colon cancer has been associated with red and processed meat intake [8]. Moreover, a dietary pattern based on high-carbohydrate intake, and high-sugar content beverages after colorectal cancer diagnosis may increase risk of recurrence and mortality after colorectal cancer diagnosis [9, 10]. Besides, folate deficiency in diet induce tumourigenesis and *MTHFR* (methylenetetrahydrofolate reductase) gene depletion produces a defect in DNA repair [11, 12]. Another factor that indirectly influences colon cancer is smoking [13–15]. Cigarette smoke induces prostaglandin E2 synthesis [16] and in high concentrations are considered a high-risk factor for colon cancer [17, 18]. Moreover, high levels of prostaglandin E2 in rectal mucosa has been directly correlated with high body mass index (BMI) [19]. Also high BMI was associated with a significantly high-risk of CRC with no or weak expression of fatty acid synthase [20] and β-catenin-negative colonic tumours [21]. Above all determination methods to diagnose obesity BMI are widespread used. Body mass index has been proposed as a ratio to predict high-risk colorectal neoplasias [22]. This association may be due to the large spectrum of cytokines and metabolites produced by adipose tissue which exhibit pro-inflammatory and cancer prone characteristics. In fact, cytokines produced by adipose tissue triggers insulin resistance [23] and mediates proliferation, migration, angiogenesis [24] and induction of oxidative stress [25]. The more influential products of oxidative stress are 4-hydroxy-2-nonenal (4-HNE) and malondialdehyde (MDA). Both compounds produced in the adipose tissue have an extraordinary effect on whole body metabolism. In this review we will highlight the relationship between obesity and CRC and the importance to consider obesity in the standard of care dealing with colorectal cancer patients.

## Review

### Methods to determine obesity in their connection with CRC

Some reports have proposed waist circumference instead of height [26, 27] for the determination of some obesity ratios. This is the case of waist-to-hip ratio [28], that seems to achieve significance with a higher CRC risk in men but not in women [29]. Other ratios are recently used to determine obesity and correlate with CRC, like impedance analysis [30], visceral fat tissue amount (VAT) [31–33] or visceral-to-subcutaneous fat ratio [34–36] (Table 1). Nevertheless, the most common ratio to diagnose obesity is by using BMI that must be 30 kg/m^2^ or greater. Still, abdominal visceral and subcutaneous adipose tissue is considered more accurate and pathogenic than BMI [37].

**Table 1:** Methods and ratios more commonly used to determine obesity

Index	Range		Ref.
Body mass index (kg/m^2^)	Underweight < 18.5		[38]
	18.5 < Normal <24.9		
	25 < Overweight <29.9		
	30 < Obesity		
Visceral adiposity ratio	Moderate	Severe	[31]
<30 years	2.59–2.73	>2.73	
≥30 < 42 years	2.54–3.12	>3.12	
≥42 < 52 years	2.17–2.77	>2.77	
≥52 < 66 years	2.32–3.25	>3.25	
≥66 years	2.42–3.17	>3.17	
Waist-to-hip ratio	Women	Men	[28]
	Normal < 0.8	Normal < 0.9	
	0.81 < Overweight < 0.84	0.91 < Overweight < 0.99	
	0.85 < Obesity	1.00 < Obesity	
Waist-to-height ratio	Women	Men	[26, 27]
	Extremely slim < 0.34	Extremely slim < 0.34	
	0.35 < Slim < 0.41	0.35 < Slim < 0.42	
	0.42 < Healthy < 0.48	0.43 < Healthy < 0.52	
	0.49 < Overweight < 0.53	0.53 < Overweight < 0.57	
	0.54 < Obesity	0.58 < Obesity	
Biolectrical impedance (% fat)	Women	Men	[30]
	28.3 ± 5.1 Normal	18.0 ± 4.5 Normal	
	35.7 ± 2.8 Overweight	23.3 ± 4.2 Overweight	
	41.6 ± 3.9 Obese	31.2 ± 5.1 Obese	
Visceral-to-subcutaneous fat ratio	Healthy (gynoid fat) < 0,4 < Risk (android fat)		[34–36]

### BMI

Body mass index, also called Quetelet index, is expressed as weight in kilograms/height in meter square [38]. A large number of studies have reported an association between high BMI and colorectal cancer. For example, a study conducted by the American Cancer Society [39] the risk associated with high BMI (above 30 kg/m^2^) was 1.8 for men and 1.2 for women compared with a BMI below 25 kg/m^2^. Other reports found a stronger association between BMI and colorectal cancer in different countries [40–43], but this association was rather controversial according to sex in other studies [44, 45]. Early life body fatness would be associated with a higher risk of developing colorectal cancer. Here, the relative risks comparing BMI categories ≥27.5–<19 kg/m^2^ were 1.44 (1.06–1.95, at age 18; P = 0.009) for women and 1.18 (0.84–1.65, at age 21; P = 0.57) for men [46].

The association between colorectal cancer risk and BMI is, in general, stronger for cancers localised in the distal colon than other localizations [43–45, 47]. Concerning rectal cancer some studies have shown scarce evidence for a connection with BMI [41, 45]. Body mass index was also related to higher risk of colon polyps or adenomas specially in male population [48].

### VAT

Visceral adipose tissue (VAT) could be easily quantified by computerized tomography [31], and has been identified as a risk factor for colorectal adenomas [49] and carcinomas [32, 50].

Although not always VAT was associated with risk of colon adenomas [51], in other studies high VAT showed a statistical significant association between proximal, multiple and advanced adenomas (p < 0.05) [49]. These findings related VAT index with the development and progression of colorectal adenoma, and it turned out to be a more appropriate obesity index for colorectal adenoma than BMI in both sexes [49]. Compared to subcutaneous adipose tissue, VAT revealed high levels of markers of inflammatory lipid metabolism and some of them associated with CRC tumour stage [50]. Concerning chemotherapy resistance VAT has been considered a poor prognostic marker in CRC receiving adjuvant chemotherapy [52]. Patients with high VAT had a significantly lower overall survival (54.8 vs 87.1 %, P  =  0.004) and disease-free survival (48.4 vs 77.4 %, P  =  0.007) if compared to patients with low VAT. Furthermore, VAT was independently associated to a reduced overall survival (HR  =  7.0; 95 % CI 2.0–24.6; p  =  0.002) [52]. But it is not always the case, other authors support low VAT could be poor prognosis marker during chemotherapy administration because it could lead to nutritional supply impairment and a subsequent malnutrition [53, 54].

However, VAT has been acknowledged to be more pathogenic than BMI [55].

## Biologic performances of obesity

### Obesity as a mutagenic factor for CRC

There are multiple molecular pathways to metabolize fat in the adipocytes. One of the mechanisms to process that fat is by peroxidation [56]. However, when cell has to manage with medium or high rates of lipid peroxidation reaction turns into toxic conditions and oxidative stress abate DNA repair capability, and then cells induce apoptosis that leads to disease [57, 58]. Two of the products of lipid peroxidation are 4-hydroxy-2-nonenal (4-HNE) and malondialdehyde (MDA). While MDA is a highly mutagenic compound, 4-HNE is basically toxic and functions as deregulators of different molecular pathways [59, 60].

4-hydroxy-2-nonenal is nowadays considered as the major bioactive marker of lipid peroxidation and a signaling molecule involved in the regulation of several transcription factors sensible to stress [61]. Some of these factors are activating protein-1 (AP-1), NF-κB, and peroxisome-proliferator-activated receptors (PPAR). Activating protein-1 transcription factor control cell proliferation, survival, and death. Growth factors, cytokines, cellular stress, and many other stimuli activate AP-1 [62]. NF-κB induce gene transcription of genes involved in the regulation of inflammation [63]. Peroxisome-proliferator-activated receptors act as key transcriptional regulators of lipid metabolism, mitochondrial biogenesis, and antioxidant defence [64]. 4-hydroxy-2-nonenal increase PPAR gene expression and accelerate adiponectin protein degradation in adipocytes [65]. Peroxisome-proliferator-activated receptors has been also reported to arrest colorectal cancer proliferation [66, 67], however it has also been linked to poor outcome in metastatic colorectal cancer (mCRC) [68]. 4-hydroxy-2-nonenal also upregulates prostaglandin E2 [69] and cyclooxygenase-2 (COX-2) [70], two factors associated with high proliferative colorectal cancer [71].

Furthermore, 4-HNE is involved in cell proliferation, differentiation, cell survival, autophagy, senescence, apoptosis and necrosis via activation of mitogen-activated protein kinases (MAPK), PIK3CA—AKT pathways, and protein kinase C. Some of the factors included in the MAPK pathway are ERK, P38, and JUN. Mitogen-activated protein kinases signaling pathway has been one of the most altered molecular mechanism in CRC [72, 73]. Interestingly, *RAS/RAF* mutations are the most commonly found alteration in colorectal cancers [74, 75].

In oxidative stress conditions, an important cellular response is the activation of the PIK3CA—AKT pathway that involves the oxidation and subsequent inactivation of *PTEN*, a tumour suppressor gene [76]. Both molecular pathways are connected by EGFR that has improved colorectal cancer classification and treatment [77].

Protein kinases C (PKCs) are a family of multifunctional enzymes that play crucial roles in the transduction of many cellular signals such as control of cell proliferation, survival, and transformation by phosphorylating various targets. Protein kinases C can also be activated by oxidative stress [78]. Moreover, oxidative stress produces a wide range of DNA mutations [79] by exogenous oxidative stimuli like high levels of alkylating agents [80], radiation [81], antioxidant depletion [82] or during inflammation process [83].

Malondialdehyde has shown to be a highly mutagenic agent in eukaryotic cells [84] and also has tumourigenic properties [85]. MDA regulated islet glucose-stimulated insulin secretion through WNT pathway [86]. Under stress conditions MDA has high ability to react with proteins or DNA that leads to the formation of adducts [87], and its aberrant expression has been associated with different pathologies [88–91]. This fact is due to the capability of MDA to react physiologically with several nucleosides (deoxy-guanosine and cytidine) to form adducts to deoxyguanosine and deoxyadenosine, resulting in pyrimido(1,2-a)purin-10(3H-)one (M1-dG) [84, 92]. In contrast, vitamins intake was associated with reduced levels of M1-dG [93] that supports the role of vitamins as a protective factor against cancer.

**Fig. 1:**
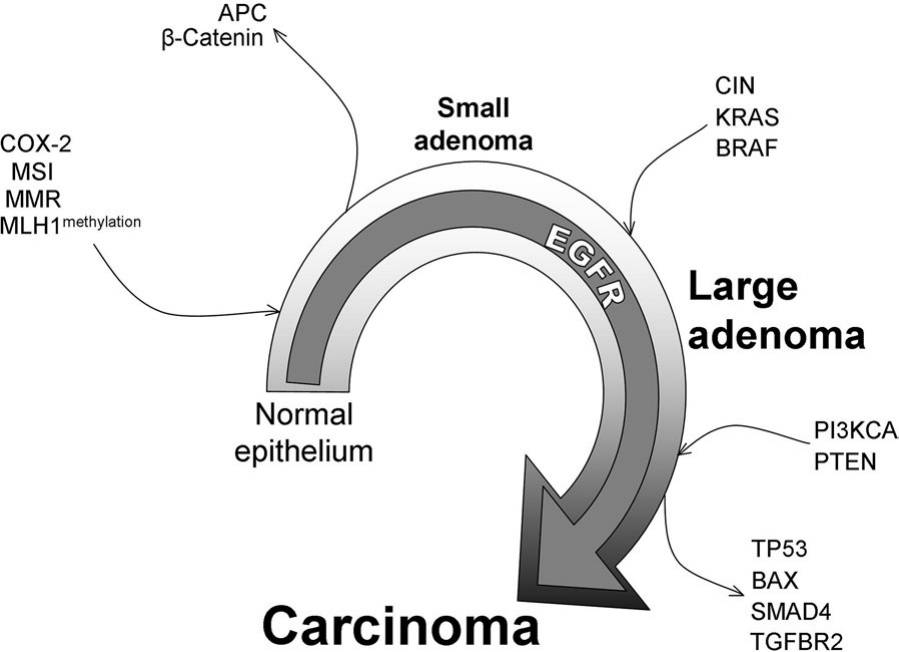
Transformation model of CRC. *Ingoing arrows* concern oncogenic effectors that are turned on in the cancer progression. *Outgoing arrows* highlight tumour suppressor factors that become inactivated in colon cancer

Accumulation of mutation in crucial factors enable the genetic process of CRC described initially by Fearon and Vogelstein and updated with experts recommendations by Rex et al. (Fig. 1) [94, 95]. Here, serrated CRC arise from adenomas in a process that in most people takes at least 10 years. The initiation of CRC involves several genetic alterations that begins with chromosomal instability (CIN), which causes numerous changes in chromosomal structure and copy number [96]. Chromosomal instability is an efficient mechanism that causes the loss of a wild-type copy of tumour-suppressor genes, such as *APC*, *TP53* or *SMAD* family member 4 (*SMAD4*) [97]. Other key event in colorectal cancer initiation is the induction of COX-2, throughout EGFR pathway [98], that mediates the synthesis of prostaglandin E2, an agent strongly associated with colorectal cancer development, stem cell expansion and metastasis [99, 100]. The most commonly found mutation in colorectal cancer involves the APC protein that leads its inactivation. In the absence of functional APC, oncogenic WNT—Catenin beta 1 (CTNNB1) pathway is activated [97, 101]. *APC* mutations is associated to familial adenomatous polyposis [102, 103]. *MLH1* confers a lifetime risk of colorectal cancer of about 80 % and the methylation status is an effective biomarker for Lynch Syndrome [104–106]. Mismatch-repair deficiency (MMR) leads the inactivation of some anti-tumour factors [107, 108] such as transforming growth factor β (TGF-β) receptor type II (TGFBR2) [109], BCL2-associated X protein (BAX), Caspase-5 and TP53 [110–113]. Furthermore, the microsatellite instability (MSI) pathway, crucial for cancer progression, is initiated by *MMR* mutation or by *MLH1* methylation [114]. Interestingly, MSI status in colonic tumours has been associated to obesity, cigarette smoking, refined carbohydrate, and red meat consumption [115–117].

Several oncogenes promote colorectal cancer development. Oncogenic mutations of *RAS* (including *NRAS* and *KRAS*) and *BRAF*, which activate the MAPK signaling pathway, occur in 37 and 13 % of colorectal cancers, respectively [118–120]. Brändstedt et al. reported statistically significant association between high waist-to-hip (WHR) ratio and BMI with an increased risk of *KRAS*-mutated colorectal tumours in men. However, in women only high WHR was significantly associated with an increased risk of *KRAS*-mutated colorectal cancers [121]. In addition, 33 % of colorectal cancers carry activating somatic mutations in *PIK3CA*, which encodes the catalytic subunit of phosphatidylinositol 3-kinase [122]. Other alterations involve loss of *PTEN* [123, 124]. EGFR is essential for CRC initiation and development which triggers MAPK and PIK3CA—AKT signaling pathway [125–128].

On the other hand, adipose tissue produces biochemical compounds involved in deregulation of whole-body metabolism and affect evolution of CRC.

### Obesity promotes deregulation of metabolism

**Fig. 2:**
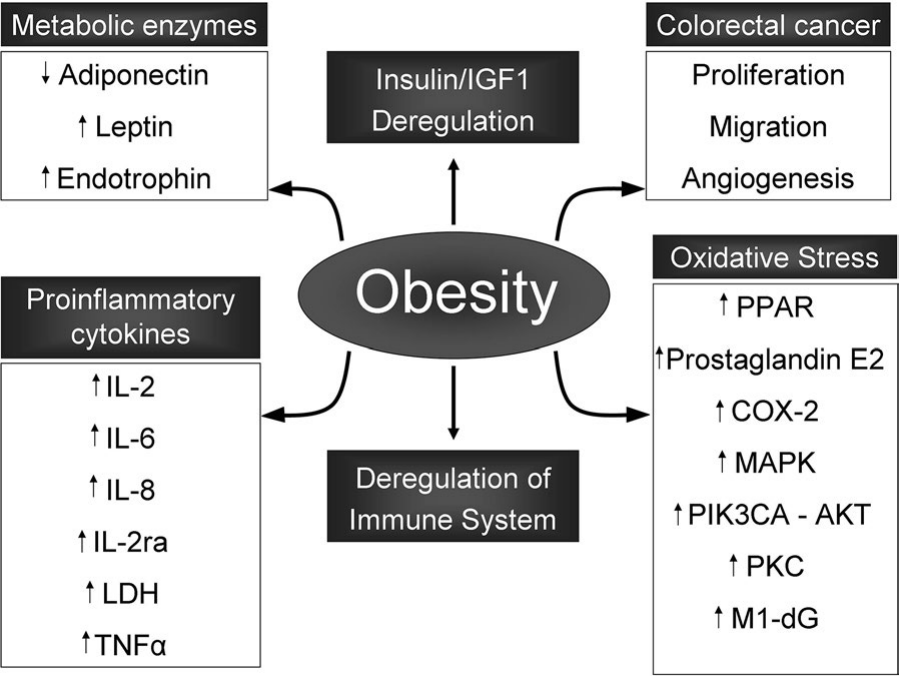
Schematic diagram of biochemical features of obesity. *Up* and *down arrows* denote up- or downregulation respectively

Obesity leads co-morbidities like diabetes, impairment in lipid metabolism and endocrinologic changes that allow cancer progression (Fig. 2) [129]. For example, the regulation of aromatase in mammary fat-tissues is interesting for clinical practise specially dealing with breast hormone-dependent cancers [130, 131]. Adipose tissues have long been identified as significant sites for steroid hormone transformation and action [132]. In addition, obese patients commonly present deregulation of insulin and/or insulin growth factor 1 (IGF1) that has also been linked to cancer [133] because insulin is considered a strong mitogen factor and stimulates DNA synthesis [134]. In fact, glucose and insulin levels exhibited statistically significant associations with colorectal cancer [135, 136] because their ability to increase the proportion of cells with metabolically active mitochondria [137]. In respect to IGF1, different studies support the role of this factor in colorectal carcinogenesis [138–140], chemoresistance [141], metastasis [142, 143] and prevention from apoptosis [144, 145].

Other obesity proteins are directly related to CRC. This is the case of adiponectin, leptin and endotrophin. High serum levels of adiponectin was inversely associated with lower BMI and waist circumference [146]. Furthermore, high level of adiponectin was significantly associated with lower risk of both colorectal adenoma and carcinoma [147]. Adiponectin (OR  =  0.50; CI: 0.3–0.8) and the soluble receptor for advanced glycation end products (sRAGE, OR  =  0.4, CI: 0.3–0.7) were inversely related to the presence of diverticulosis [148].

Leptin, also called obese gene product, regulates body weight and fat deposition through effects on metabolism, and increases appetite [149, 150]. Hyperleptinemia is characteristic of obese patients and leptin has been reported to promote colon carcinogenesis [151]. In fact, in vitro models of leptin stimulation showed not only increased proliferation of CRC cell lines [152] but also angiogenesis and production of other cytokines like IL-6 [153]. In humans patients, high leptin levels have been associated with higher risk to develop adenoma in obese individuals (BMI > 30) compared to lean individuals (BMI < 25) [154]. In contrast, other approaches suggest that leptin may be associated with risk of colorectal adenomas in men, however it does not correlate in women [155]. High leptin levels were also positively associated with diverticulosis (OR  =  5.5, CI: 2.0–14.7) [148]. Some genes that regulate the WNT—β-catenin pathway such as *MDM2*, *PIK3R1*, and *RB1* were upregulated by leptin. Importantly, leptin supplementation induced expression of IGF-mediated pathway genes and their products such as IGFBP-6, IGF1, and Crim1 [153]. On the contrary, other factors were downregulated like IGFBP-2, IGFBP-3, IGFBP-4, IGFBP-5, and Nov [153].

Endotrophin is the result of cleaved form of C5 fragment of the collagen type VI alpha 3 chain (COL6A3) [156]. It seems that it acts as a signaling molecule modulating several effects in the tumour-stromal environment like epithelial-mesenchymal transition of cancer cells, fibrosis, angiogenesis and immune cell infiltrations [156], especially observed in breast cancer [157].

Thus, obesity changes the stability and homeostasis of adipocytes and produces different hormones and cytokines leading to inflammation, a process that joins obesity and CRC initiation and progression.

### Obesity and inflammation: a direct link with CRC

Obesity is a state of low-grade chronic inflammation and is closed linked to cancer (Fig. 2) [158]. It is probably because the largest endocrine organ in the body is the adipose tissue and it stimulates secretion of several signaling cytokines. Some of the cytokines produced by adipose tissue are IL-8, IL-6, IL-2, lactate dehydrogenase (LDH), tumour necrosis factor alpha (TNFα), as well as IL-2 receptor alpha (IL-2ra) (Fig. 2). These cytokines have been reported to play a role not only in both tumour initiation and progression [159–161] but also in promoting epithelial mesenchymal transition and metastasis in obese patients [162, 163].

For example, high levels of LDH, IL-2ra and IL-8 are considered poor prognostic factors in obese metastasic CRC patients [164]. Other cytokines like PDGF, TGF-β, FGF and VEGF have been found to be upregulated in the mammary-associated fat in humans [24].

IL-6 family of cytokines is highly upregulated in many cancers and it is considered as one of the most important cytokine families during tumourigenesis and metastasis [165]. Tumour necrosis factor alpha promote insulin resistance in the liver and other metabolism-controlling organs with the subsequent cancer promotion [24].

Also proinflammatory factors, like interferon gamma-inducible protein-10 (IP-10), function as chemo-attractant to enhance local inflammation [166]. Interestingly, IP-10 expression was significantly associated with increased likelihood to develop adenomas while TNFα showed a trend (P = 0.09) [146].

On the other hand, when tumour is initiated it may arrest inflammation to escape of immune cells. One mechanism to assess this is by decreasing the inflammation signal of adipose tissue through the regulation of macrophages phenotype. Other cytokines are involved in such process like interferon gamma (IFN-γ) and IL-10 that are produced by tumour microenvironment [167]. This cytokines keep M2 polarization of macrophages and those with M1 phenotype are converted into M2 [168]. M2 macrophages are those activated under lean conditions [169] and associated to tumour progression [170]. Some B cells (B1) could also induce macrophages phenotypic change [171–173]. Moreover, in response to upregulation of IFN-γ and IL-10, cells from tumour microenvironment like antigen presenting cells (APCs), tumour associated myeloid derived suppressor cells (MDSC), and M2 macrophages are able to produce indolamine 2,3-dioxygenase (IDO). This compound promotes anti-inflammatory phenotype by suppression of T and NK cell activity which enables immune escape of cancer, angiogenesis and metastasis [174–176].

## Discussion

Over the past few decades, obesity has emerged as a global epidemic. A cross-sectional survey conducted in 2003–2004 revealed that 66.2 % of United States adults who averaged 20-and-74 years old were either overweight or obese [177]. Nowadays, in the US more than 30 % of the population is obese [1] and a thin line separates obesity and colorectal neoplasia. The most common ratio to classify individuals according anthropometric measures is BMI [178]. Wu et al. reported that obese patients appear to have worse overall survival than normal-weight patients with CRC [145]. Studies concerning chemotherapy resistance are rather controversial, on the one hand, it is showed an association between high BMI and VAT with reduced time-to-progression after receiving first-line anti-angiogenic therapy [179]. On the other, anthropometric measures are considered poor outcome markers since it reveals a nutritional impairment during chemotherapy administration [53, 54]. Other study associated BMI with CRC-related mortality in pre-diagnosis men [6] however BMI association with CRC outcome according to sexes is rather disputed. Therefore, the standard use of BMI as indicator of obesity may be examined. The National Health and Nutrition Examination Survey (NHANES) reported >50 % of individuals with high body fat content would be classified as being normal of just overweight [180]. It seems that correlation between cancer and BMI is far to be linear because not all obese patients might eventually develop colorectal cancer. However, a recent report highlights BMI as an independent predictor for overall survival dealing with overweight and obese patients with mCRC treated with Bevacizumab [181]. Additionally, the association of obesity with survival of CRC patients is likely to be affected by the timing of BMI measurement [182]. An obese prediagnostic measured by BMI months–years before presentation of CRC has been consistently linked to worse survival [6, 183]. All these results suggest that perhaps BMI has enough limitations to be considered as the best marker of obesity, particularly individuals in the intermediate BMI ranges, men and the elderly. Therefore, in the near future, find a marker to highlight obesity to tailor screening practices for CRC will be a challenge in this field.

There are several factors that influence weight gain but are mostly diet and physical activity. A diet based on different meats, poultry, fish and eggs was associated with a 50 % increase in risk among men [184]. Moreover, it has been reported how high fat content diet, increase intestinal cancer in genetically susceptible mice [185]. By contrast, vegetable fiber intake has been demonstrated to offer a protective effect on CRC [186]. Thus, a dietary habits change becomes a crucial element to reduce potential risk.

The link between physical activity and cancer is controversial. Although it has been estimated in US that 13–14 % of colon cancer may be attributable to physical inactivity [184], other reports showed body weigh loss was not enough to reduce cancer risk [187]. The lack of physical activity can lead to adipose tissue accumulation and the subsequent stimulation of inflammatory cytokines that could promote CRC [188].

Other crucial factor to consider is gut microbiota. Colonic tumours revealed enrichment in fusobacteria what has been visualized by FISH and detected by PCR [189, 190]. Moreover, this kind of microorganisms have been related to those CRC carrying MSI, *TP53* wild-type, CIMP positive, *MLH1* positive methylation status, and *CHD7/8* mutation positive [191]. Other authors demonstrate involvement of *fusobacterium* with high-grade dysplasia [190] and recently with poor prognosis CRC [192]. *Fusobacterium* also promotes downregulation of antitumour CD3 + T cell–mediated adaptive immunity [193].

Other factors play an essential role in hematopoietic and chemotactic functions to promote tumour initiation, growth and metastasis and may limit survival in patients with CRC [194–196]. Elevated levels of some inflammatory cytokines contributed to poor survival rates in mCRC, especially interleukin 8 (IL-8), and LDH [197]. The association between IL-8 and LDH levels and risk of death is higher in obese mCRC patients than in non-obese mCRC patients. On the other hand, high levels of adiponectin present low-risk of CRC [198].

The chronic low-grade inflammation state produced by obesity leads the induction of oxidative stress factors. The major bioactive product of lipid peroxidation is 4-HNE and it is responsible of deregulation of multiple pathways involved in cell proliferation differentiation, cell survival, autophagy, senescence, apoptosis and necrosis. The molecular pathways mainly altered by 4-HNE includes MAPK, PIK3CA—AKT and NF-κB, in addition to accelerating adiponectin degradation and upregulation of prostaglandin E2, all them related to CRC development. Moreover, accumulation of DNA mutations, as in *APC*, *KRAS*, *NRAS*, *BRAF*, or *PIK3CA,* and insulin secretion sets obesity as a multifactor phenomenon involved in CRC initiation and aggressive development. These events may be fostered by MDA, the other bioreactive and mutagenic product of lipid metabolism. To dissect how outer effects affect transformation, initiation, and progression of CRC, a new field has emerged called “molecular pathologic epidemiology”. This domain will provide future scientific explanations of tumorigenesis related to life style, diet and other environmental factors, in order to bring new prevention strategies [199, 200].

These facts point out the strong involvement between obesity and CRC, even if it seems to be higher for colon than for rectal neoplasias [201].

## Conclusions

As we have seen, one of the main diseases in western countries is obesity. Over the last decade several studies found a close connection between obesity and development of colorectal neoplasias. Then, biochemical studies revealed that oxidative stress conducted by lipid peroxidation produce secondary metabolites with highly mutagenic and toxic properties. These products lead the initiation of pathological disorders by accumulation of genomic aberrations. Development of CRC and evolution to malignant phenotype is allowed by accumulation of additional genomic aberrations that may be assisted by these high mutagenic products. Therefore, obesity performs a complex biological activity regulation like cytokines production leading insulin resistance or deregulation of IGF1 and immune system among others. Then, obesity must be diagnosed and controlled not only to prevent comorbidities like high blood pressure, high levels of triglycerides, cholesterol or elevated fasting plasma glucose, but also obesity must be considered as an early warning for a potential pre-neoplasic lesions.

## Abbreviations

APCs: antigen presenting cells
AP-1: activating protein-1
BAX: BCL2-associated X protein
CTNNB1: β-catenin
BIA: bioelectrical impedance analysis
BMI: body mass index
CIN: chromosomal instability
COL6A3: collagen type VI alpha 3
CRC: colorectal cancer
CT: computerized tomography
CIMP: CpG island methylator phenotype
EGFR: epidermal growth factor receptor
FISH: fluorescent in situ hybridization
HR: hazard ratio
4-HNE: 4-hydroxy-2-nonenal
IL-2ra: IL-2 receptor alpha
IDO: indolamine 2,3-dioxygenase
IGF1: insulin growth factor 1
IFN-γ: interferon gamma
IP-10: interferon gamma-inducible protein-10
LDH: lactate dehydrogenase
MDA: malondialdehyde
mCRC: metastatic colorectal cancer
MTHFR: methylenetetrahydrofolate reductase
MSI: microsatellite instability
MMR: mismatch-repair deficiency
MAPK: mitogen-activated protein kinase
OR: odds ratio
PPAR: peroxisome-proliferator-activated receptors
PIK3CA: phosphoinositide 3-kinase
PKC: protein kinase C
M1-dG: Pyrimido(1,2-a)purin-10(3H-)one
sRAGE: soluble receptor for advanced glycation end products
S/T ratio: skinfold thickness
TGFBR2: transforming growth factor β (TGF-β) receptor type II
MDSC: tumour associated myeloid derived suppressor cells
TNF-α: tumour necrosis factor
VAT: visceral adipose tissue
WHR: waist-to-hip
